# Detection of Transmitted Power Violation Based on Geolocation Spectrum Database in Satellite-Terrestrial Integrated Networks

**DOI:** 10.3390/s20164462

**Published:** 2020-08-10

**Authors:** Ning Yang, Pinghui Li, Daoxing Guo, Linyuan Zhang, Guoru Ding

**Affiliations:** 1 College of Communications Engineering, Army Engineering University, Nanjing 210007, China; ningyang0928@163.com (N.Y.); lph8210@126.com (P.L.); dr.guoru.ding@ieee.org (G.D.); 2 Jiangnan Institute of Computing Technology, Wuxi 214083, China; zhanglinyuan5@163.com

**Keywords:** satellite communication, spectrum sharing, geolocation spectrum database, generalized likelihood ratio test

## Abstract

This paper investigates the detection of the transmitted power violation (TPV) in the satellite-terrestrial integrated network, where the terrestrial base station may break the spectrum policies so that severe damages are made to the satellite systems. Due to the lack of prior information on specific abnormal behaviors, this problem is complex and challenging. To tackle it, we first turn to the geolocation spectrum database based detecting framework, where not only the tasks of each segment but also the spectrum policies are specified. Then, the ternary hypothesis test and the generalized Neyman–Pearson (GMNP) test criterion are applied to maximize the detection probability under the false-alarm constraint. What is more, the Abnormal after Normal (AaN) detector is developed to simplify the analysis. Finally, simulations are conducted to demonstrate that the proposed detector can realize the detection of TPV in most cases at the expense of less than 10% detection probability.

## 1. Introduction

### 1.1. Background and Motivation

With the rapid development of satellite communication, the satellite-terrestrial integrated network has become a new way to enhance the spectrum efficiency [1,2,3,4,5,6,7,8]. However, in the satellite-terrestrial integrated network, the terrestrial networks from different sources have different requirements and diversified states, which is very easy to breed abnormal spectrum using behavior. The order of spectrum usage is seriously disrupted. Therefore, to maintain the order of the spectrum, it is necessary to monitor the abnormal spectrum behavior in the satellite-terrestrial integrated network.

Spectrum policies are designed to achieve full-spectrum use with acceptable interference by the geolocation spectrum database [9,10,11,12]. However, spectrum usage can be optimally realized only by strict implementation of spectrum policies. Considering malicious attacks, selfish motivations, equipment failures, and many other factors, the spectrum order may be attacked by spectrum misuse [13], such as illegal access, abnormal power transmission, etc. TPV is a typical abnormal spectrum behavior, which has many inducing factors, resulting in the terrestrial base station transmitting power deviating from the established transmitting power. From the perspective of selfish drive or equipment failures, the illegal behaviors of TPV can be divided into the following two categories.

One is aggressive power transmission; to achieve a higher data rate and improve their own benefits or the equipment is out of order, the selfish or faulty terrestrial base station may violate the limits of transmitted power and work at a higher transmitted power level. However, the satellite earth station and other terrestrial base stations may be subject to more interference, and consequently, the overall performance of the network shows a downward trend. The other is passive power transmission; this occurs when terrestrial stations operate with lower power than allocated. According to Shannon’s capacity formula [14], the result of this behavior is a decrease in the transmission rate. As a result, it takes longer to transmit the specified content, and the extended transmission time allows the ground station to obtain an exaggerated duty cycle. Therefore, it can apply to the geolocation spectrum database for more spectrum access opportunities [15]. At the same time, it prevents other terrestrial base stations from obtaining the spectrum access opportunities which they deserve. When it comes to the detection of the TPV, the existing studies mainly focus on the detection in the scenario of terrestrial spectrum sharing rather than terrestrial-satellite spectrum sharing, however, considering the appearance of satellite signal, which may bring extra difficulty to the detection. What is worse, in the scenario of terrestrial-satellite spectrum sharing, aiming at protecting the spectrum order, the existing work mainly focuses on the available transmitted power level rather than the detection of TPV. Motivated by the above observation, it is of great importance to detect the TPV in the scenario of terrestrial-satellite spectrum sharing to prevent this relatively covert behavior from jeopardizing the whole network.

In general, in the satellite-terrestrial integrated network, the terrestrial network can apply for spectrum resources from the satellite system with the help of the geolocation spectrum database to realize satellite-terrestrial spectrum sharing. However, when the transmission power of the terrestrial base station is abnormal, it may affect the normal work of other terrestrial base stations or satellite earth stations, or hinder the normal application of other ground base stations to access, and eventually reduce the spectrum efficiency of the whole network.

### 1.2. Related Work

Spectrum policies are under the premise that the geo-spectrum database authorizes the users who have a requirement to utilize the spectrum. What is more, no business can be conducted on these premises lest incurring heavy penalties. However, there always will be some users who may break these rules to improve their own benefits and further cause severe damages to the authorized user. Existing studies about the detection of the TPV mainly concentrate on the joint detection and estimation of spectrum misuse [16,17,18,19]. In ref. [16], generalized multi-hypothesis Neyman–Pearson (GMNP) criterion is utilized to detect the illegal access in the terrestrial networks where violating the spectrum access schedule is defined as opportunistic illegal access. In ref. [17], a hybrid binary hypothesis test is adopted to detect the abnormal power emission of the secondary user and further ensure the communication of other secondary users rather than the primary user. In ref. [18], aiming at determining the legitimacy of the working secondary users, a new cooperative spectrum sensing scheme is proposed to solve this problem in the presence of a primary user emulation attack (PUEA). In ref. [19], outage probability which can reflect the impact of secondary transmissions on the primary receiver is used to evaluate the performance of the spectrum sensing techniques. Note that in those studies, the spectrum sharing scenario mainly focuses on terrestrial networks rather than satellite-terrestrial integrated networks.

While in the scenario of satellite-terrestrial integrated networks, the authors propose the cognitive satellite-terrestrial radios for satellite-terrestrial integrated systems [20] for the first time. In ref. [8], aiming at protecting the communication of the satellite, the concept of the protected area is proposed by means of accumulating the interference of terrestrial base stations, and furthermore, the protection radius is deduced. According to the interference power level, the models of interference in the satellite-terrestrial integrated networks have been formulated in ref. [21]. The available transmitted power levels are figured out by analyzing the interference between geostationary (GEO) satellite systems and non-geostationary (NGEO) satellite systems, where the GEO satellite is regarded as the primary user and NGEO satellite is regarded as the secondary user, respectively [7]. Note that, however, some studies analyze the available transmitted power levels but few on detection of the TPV in the satellite-terrestrial integrated networks.

To the best of the authors’ knowledge, there are no existing studies considering the detection of TPV in the satellite-terrestrial integrated networks. In the scenario of satellite-terrestrial spectrum sharing, the existence of satellite signals, which interferes with the detection of TPV, bringing great difficulties to the distinction of abnormal signals from normal signals. However, to protect the communication of the whole network, it is of great importance to propose and study the behavior of TPV.

### 1.3. Contributions

In this paper, we investigate the geolocation spectrum database-based detection of TPV in a satellite-terrestrial integrated network, where every sensing node allocated in this network collects and processes the sensing data. In particular, the main contributions of this paper can be summarized as follows:

- Formulate a generalized model for spectrum sharing in the satellite-terrestrial integrated networks, where the terrestrial base station can apply to the geolocation spectrum database for the available spectrum of the satellite.
- Propose a detection framework based on geolocation spectrum database, which specifies the tasks and roles of the satellite-terrestrial integrated network, surveillance network, and geolocation spectrum database no matter when the terrestrial base station operates at the single power level or multiple power level.
- Analyze the optimization problem under both single and multiple transmitted power level scenarios, and further the AaN detector is derived.
- Provide comprehensive simulations under various parameter configurations, which represent that the superiority and effectiveness of the proposed detecting framework.

The remainder of this paper is organized as follows. The system model of TPV is formulated in Section 2. Section 3 derives the test statistics under both single and multiple transmitted power levels. Simulation is given in Section 4, followed by the conclusion in Section 5.

## 2. System Model

### 2.1. Database-Based Detection Framework

**Figure 1:**
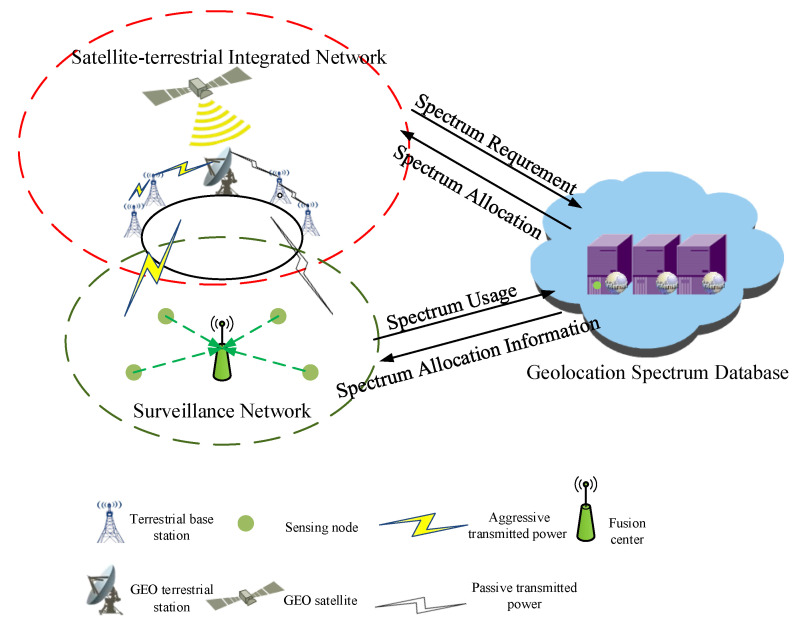
The generalized detection model for the transmitted power violation (TPV) in the downlink scenario of satellite-terrestrial spectrum sharing, which consisted of satellite-terrestrial integrated network, geolocation spectrum database, and surveillance network.

In this paper, terrestrial base stations are considered to apply for the downlink spectrum opportunities from satellite networks through the geolocation spectrum database, as shown in Figure 1.

Firstly, the terrestrial base station forwards the spectrum usage requirements to the geolocation spectrum database, and then the geolocation spectrum database allocates the corresponding spectrum resources and formulates the corresponding spectrum utilization rules. At the same time, the geolocation spectrum database shares the spectrum utilization information of the spectrum users which includes the allocated channel, the optional transmitted power levels, and the working state of satellite with surveillance network. Next, the spectrum surveillance network distributes the monitoring task to the sensing nodes by combining the monitoring target and the relative positions of the sensing nodes. Each sensing node monitors the allocated channel, determines whether the target user violates the spectrum using rules according to the observation of a certain time slot, and reports the judgment result to the fusion center. Finally, according to the reported data of the sensing nodes, the fusion center feedbacks the detection result of the TPV after the integration and the analysis of the data to the management center.

### 2.2. Hybrid Hypothesis Testing Model

For normal spectrum usage, when the satellite (PU-T) is working, the channel is occupied by the satellite signal ($N_{0}$) or the terrestrial base station (SU-T) with the permitted L level transmitted power ($N_{l}$), and the spectrum sharing state is expressed as follows:

(1) $$\left\{\begin{array}{l} N_{0}:y_{k}\left(m\right)=h_{0,k}\sqrt{P_{0}}s_{k}\left(m\right)+w_{k}\left(m\right),\\ N_{l}:y_{k}\left(m\right)=h_{0,k}\sqrt{P_{0}}s_{k}\left(m\right)+h_{1,k}\sqrt{P_{t}^{\left(l\right)}}s_{k}\left(m\right)+w_{k}\left(m\right),l=1,2,\ldots ,L, \end{array}\right.$$

where $y_{k}\left(m\right)$ is the sampling of the m-th signal received by the k-th sensing node, m=1,2,…,M, *M* denotes the number of samples, $h_{0,k}$ is the gain from the satellite to the k-th sensing node, and $h_{1,k}$ is the channel gain from the terrestrial base station to the k-th sensing node, $P_{t}^{\left(l\right)}$ represents the l-th transmitted power level of L power levels, $w_{k}\left(m\right)∼N\left(0,\sigma_{w}^{2}\right)$ denotes the Gaussian white noise, $s_{k}\left(m\right)$ is the sampling of transmitted signals (normalized to unit power), i.e., $s_{k}\left(m\right)∼N\left(0,1\right)$.

**Figure 2:**
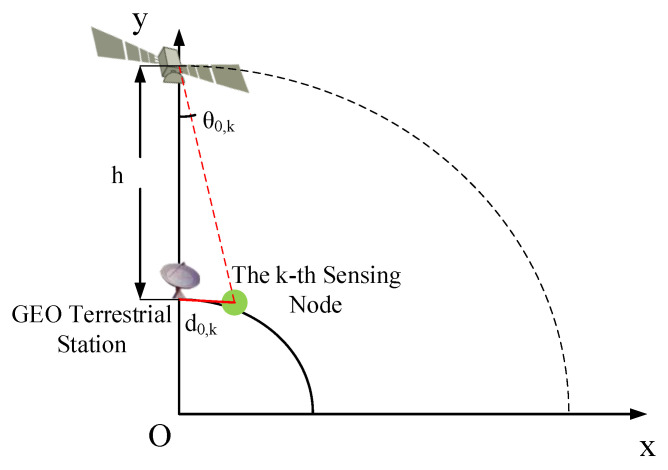
The off-axis angle of the geostationary (GEO) satellite in the direction of the k-th sensing node, $\theta_{0,k}$.

$P_{0,k}$ denotes the signal received power of the primary user at the k-th sensing node, satisfying [22]

(2) $$P_{0,k}=P_{0}h_{0,k}\left(\theta_{0,k}\right){\left(\frac{c}{4\pi f_{c}d_{0,k}}\right)}^{2}10^{\frac{A_{g}}{10}}10^{\frac{A_{c}}{10}},$$

where $P_{0}$ represents the transmitted power of the primary user (satellite), and $h_{0,k}\left(\theta_{0,k}\right)$ represents the antenna gain of the satellite at the direction of $\theta_{0,k}$, with $d_{0,k}$ as the distance between the transmitter of the primary user and the k-th sensing node, with propagation factor $A_{g}$ as gaseous absorption and $A_{c}$ as cloud or fog attenuation [23,24,25]. $A_{g}$ can be further given by $A_{g}=A_{o}+A_{w}=0.182fN^{\prime \prime }\left(f\right)$, where $A_{o}$ and $A_{w}$ are the specific attenuations due to water vapor and dry air, respectively, and the imaginary part of the frequency-dependent complex refractivity $N^{\prime \prime }\left(f\right)$ is referred to [26]. What’s more, the specific attenuation within a cloud or fog $A_{c}$ [27] can be represented as $A_{c}=K_{l}C$, with $K_{l}=0.89f/\left[\varepsilon^{\prime \prime }\left(1+\eta \right)\right]$ as the specific attenuation coefficient $\eta =\left(2+\varepsilon^{\prime }\right)/\varepsilon^{\prime \prime }$, $\varepsilon^{\prime }$ and $\varepsilon^{\prime \prime }$ denote are the imaginary part and real part of the dielectric permittivity ε of water *C* represents liquid water density in the cloud or fog. As shown in Figure 2, $\theta_{0,k}$ is the off-axis angle of the GEO satellite in the direction of the k-th sensing node and can be further calculated by $\theta_{0,k}=\arctan\left(\frac{d_{0,k}}{h}\right)$. Moreover, the GEO satellite antenna gain $h_{0,k}\left(\theta_{0,k}\right)$ refers to the standard in ITU-R.S.672-4 [28] can be described as:

(3) $$h_{0,k}\left(\theta_{0,k}\right)=\left\{\begin{array}{l} h_{s,\max}-3{\left(\frac{\theta_{0,k}}{\theta_{s,b}}\right)}^{2},\;\;\;\;\;\;\;\;\theta_{s,b}<\theta_{0,k}<b\theta_{s,b},\\ h_{s,\max}+L_{n},\;\;\;\;\;\;\;\;\;\;\;b\theta_{s,b}<\theta_{0,k}<c\theta_{s,b},\\ h_{s,\max}+L_{n}+20-25\log\left(\frac{\theta_{0,k}}{\theta_{s,b}}\right),\;\;\;c\theta_{s,b}<\theta_{0,k}<\theta_{1},\\ 0,\;\;\;\;\;\;\;\;\;\;\;\;\;\theta_{1}<\theta_{0,k}<90^{\circ },\\ 3,\;\;\;\;\;\;\;\;\;\;\;\;\;90^{\circ }<\theta_{0,k}<180^{\circ }, \end{array}\right.$$

where $h_{s,\max}$ is the maximum gain of the GEO satellite antenna, $\theta_{s,b}$ is the one half the 3 dB beamwidth, $L_{n}$ is the desired side-lobe level relative to peak gain. $\theta_{1}$ is the value of $\theta_{0,k}$, when Equation (4) is valid. For $L_{n}=-20$, the values of *b* and *c* are 2.58 and 6.32, respectively.

(4) $$\begin{array}{c} h_{0,k}\left(\theta_{0,k}\right)=h_{s,\max}+L_{n}+20-25\log\left(\frac{\theta_{0,k}}{\theta_{s,b}}\right)=0\;\mathrm{dB}. \end{array}$$

Quite different from the conventional terrestrial heterogeneous networks, $h_{0,k}\left(\theta_{0,k}\right)$ represents the characteristics of satellite communication, while the antenna of the transmitter in terrestrial heterogeneous networks is the omni-directional antenna, that is, the gain is always constant.

It is important to note that there is only one option for normal transmitted power, namely L=1, in most of the literature. However, users may work as multiple discrete transmit power levels, i.e., L≥2, in practice. Therefore, the diversity of the normal state of the spectrum will make the detection of TPV more difficult. What is worse, in the actual usage of the spectrum, there are a variety of uncertain factors, such as equipment failure, selfish drive, malicious attack, etc., which make the transmission power abnormal. As a result, there are abnormal use states in addition to normal use states of the above spectrum usage.

(5) $$\begin{array}{c} A:y_{k}\left(m\right)=h_{0,k}\sqrt{P_{0}}s_{k}\left(m\right)+h_{1,k}\sqrt{P_{a}}s_{k}\left(m\right)+w_{k}\left(m\right),P_{a}\notin \left\{P_{t}^{\left(0\right)},P_{t}^{\left(1\right)},\ldots ,P_{t}^{\left(L\right)}\right\}, \end{array}$$

where $P_{a}$ denotes the abnormal transmitted power, $P_{t}^{\left(0\right)}=0$ represents only PU is working ($H_{0}$). The deviation of $P_{a}$ and the permitted power level which can be measured denotes the degree of abnormality of the transmitted power.

It is necessary to distinguish the abnormal use state from the normal use state to realize the orderly spectrum use in the satellite and terrestrial integrated network. The detection problem can be modeled as the following mixed binary hypothesis test problem:

(6) $$\left\{\begin{array}{c} \begin{array}{c} O_{0}:y_{k}\left(m\right)=h_{0,k}\sqrt{P_{0}}s_{k}\left(m\right)+h_{1,k}\sqrt{P_{n}}s_{k}\left(m\right)+w_{k}\left(m\right),P_{n}\in \left\{P_{t}^{\left(0\right)},P_{t}^{\left(1\right)},\ldots ,P_{t}^{\left(L\right)}\right\}, \end{array}\\ \begin{array}{c} O_{1}:y_{k}\left(m\right)=h_{0,k}\sqrt{P_{0}}s_{k}\left(m\right)+h_{1,k}\sqrt{P_{a}}s_{k}\left(m\right)+w_{k}\left(m\right),P_{a}\notin \left\{P_{t}^{\left(0\right)},P_{t}^{\left(1\right)},\ldots ,P_{t}^{\left(L\right)}\right\}, \end{array} \end{array}\right.$$

where null hypothesis $O_{0}$ contains all the normal spectrum states $N_{0},N_{1},\ldots ,N_{L}$. The alternative hypothesis $O_{1}$ is equal to A, where $P_{a}$ distributes over a certain range. However, $P_{n}$ contains a lot of cases. Both $O_{0}$ and $O_{1}$ are composite consequently. At present, most detection algorithms focus on the test problem under the simple null hypothesis, while few studies involve multiple discrete components. The uncertain normal state and unknown abnormal power increase the difficulty of detection, especially in the above problems. Therefore, the current method cannot be directly applied to the problem in Equation (6). Based on the above observations, it is necessary to design an effective detector for the mixed binary hypothesis testing problem. What needs to be pointed out, in particular, is that there are two possible errors, false alarm, and omission, in the process of detecting the TPV. Specifically, as for false alarm, misjudging the existence of violations may cause the target users to suffer undeserved punishment, and the omission of violations will encourage the offending users, which will seriously weaken the efficiency of spectrum usage. Therefore, it is necessary to reduce the occurrence probability of two kinds of errors, that is, to reduce the probability of missed detection and false alarm. However, there is a trade-off between missed detection probability and false alarm probability. Next, according to the compromise problem, the detection criteria under different performance requirements are analyzed, and the corresponding detection methods are derived.

## 3. Detection of TPV under Heterogeneous False Alarm Constraint

### 3.1. Detection Criteria under Heterogeneous False Alarm Constraint

Before expanding to the multi-power level scenario, this subsection considers the detection of abnormal transmission power behavior at the power level L=1. Therefore, the question can be reformulated as:

(7) $$\left\{\begin{array}{c} \begin{array}{c} O_{0}:y_{k}\left(m\right)=h_{0,k}\sqrt{P_{0}}s_{k}\left(m\right)+h_{1,k}\sqrt{P_{n}}s_{k}\left(m\right)+w_{k}\left(m\right),P_{n}\in \left\{0,P_{t}^{\left(1\right)}\right\}, \end{array}\\ \begin{array}{c} O_{1}:y_{k}\left(m\right)=h_{0,k}\sqrt{P_{0}}s_{k}\left(m\right)+h_{1,k}\sqrt{P_{a}}s_{k}\left(m\right)+w_{k}\left(m\right),P_{a}\notin \left\{0,P_{t}^{\left(1\right)}\right\}. \end{array} \end{array}\right.$$

Considering the difference in sensitivity of different normal states to abnormal behaviors in some scenarios, it is necessary to differentiate performance constraints. Therefore, the optimization objective adopted in this section can be expressed as:

(8) $$\begin{array}{l} \max\mathrm{Pr}\left(O_{1}|O_{1}\right),\\ s.t.\mathrm{Pr}(\left.O_{1}|O_{0},P_{n}=0\right)\leq \alpha_{0},\mathrm{Pr}\left(O_{1}|O_{0},P_{n}{=P}_{t}^{\left(l\right)}\right)\leq \alpha_{1}, \end{array}$$

where, $\alpha_{0}$ and $\alpha_{1}$ are false alarm constraint constants, $\mathrm{Pr}\left(O_{i}|O_{j}\right)$ represents the probability of being judged as $\alpha_{i}$ when $\alpha_{j}$ is true. To facilitate the subsequent expression, the above problems can be equivalent to the Ternary Hypothesis test problem as follows:

(9) $$\left\{\begin{array}{l} H_{0}:\sigma^{2}=\sigma_{0}^{2},\\ H_{1}:\sigma^{2}=\sigma_{1}^{2},\\ H_{2}:\sigma^{2}\in \left(\sigma_{0}^{2},\sigma_{1}^{2}\right)\cup \left(\sigma_{1}^{2},+\infty \right), \end{array}\right.$$

where $\sigma_{0}^{2}=\sigma_{\omega }^{2}+P_{0}h_{0,k}^{2}$, and $\sigma_{1}^{2}=\sigma_{\omega }^{2}+P_{0}h_{0,k}^{2}+h_{1,k}^{2}P_{t}^{\left(1\right)}$. Therefore, the optimization objective in Equation (8) can be reformulated as:

(10) $$\begin{array}{l} \max\mathrm{Pr}\left(H_{2}|H_{2}\right),\\ s.t.\mathrm{Pr}(\left.H_{2}|H_{0},P_{n}=0\right)\leq \alpha_{0},\mathrm{Pr}\left(H_{2}|H_{1},P_{n}{=P}_{t}^{\left(l\right)}\right)\leq \alpha_{1}, \end{array}$$

where $\mathrm{Pr}\left(H_{2}|H_{j}\right),j\in 0,1,2$ represents the probability of being judged to be $H_{2}$ if the actual state is $H_{1}$.

### 3.2. Hybrid Hypothesis Testing Model

#### 3.2.1. Single-Node Detection under Heterogeneous False-Alarm Constraint

Noted that this is an optimal problem with multiple constraints, so the Lagrange multiplier method is adopted to establish the objective function as follows:

(11) $$\begin{array}{l} F=\mathrm{Pr}\left(H_{2}|H_{2}\right)-\sum_{j\in (0,1)}\rho_{j}\left(\mathrm{Pr}\left(H_{2}|H_{j}\right)-\alpha_{j}\right)\\ \;=\int_{C}p\left(y;H_{2}\right)dy-\sum_{j\in (0,1)}\rho_{j}\left(\int_{C}p\left(y;H_{j}\right)dy-a_{j}\right)\\ \;=\int_{C}\left(p\left(y;H_{2}\right)-\sum_{j\in [0,1)}\rho_{j}p\left(y;H_{j}\right)\right)dy+\sum_{j\in (0,1)}\rho_{j}\alpha_{j}, \end{array}$$

where $y=[y_{k}\left(1\right),y_{k}\left(2\right),\ldots ,y_{k}\left(M\right)]$, and *C* is the judgment domain that determines the existence of violation behaviors, satisfying $\mathrm{Pr}\left(H_{2}|H_{i}\right)\overset{\Delta }{\;=}\int_{y\in C}p\left(y;H_{i}\right)dy$. Therefore, to maximize $\mathrm{Pr}\left(H_{2}|H_{2}\right)$, as for any y∈C, $p\left(y;H_{2}\right)-\rho_{0}p\left(y;H_{0}\right)-\rho_{1}p\left(y;H_{1}\right)>0$, i.e.,

(12) $$\begin{array}{l} f\left(y\right)=\sum_{i\in \{0,1\}}\rho_{i}\frac{p\left(y;H_{i}\right)}{p\left(y;H_{2}\right)}\\ \;\;=\sum_{i\in \{0,1\}}\rho_{i}{\left(\frac{\sigma_{2}^{2}}{\sigma_{i}^{2}}\right)}^{\frac{N}{2}}\exp\left(\frac{\sum_{n=0}^{N-1}y_{n}^{2}}{2\sigma_{2}^{2}}-\frac{\sum_{n=0}^{N-1}y_{n}^{2}}{2\sigma_{i}^{2}}\right)<1, \end{array}$$

where $\sigma_{2}^{2}$ is the variance of observed data in the case of TPV ($H_{2}$). The test statistic is $T=\sum_{n=0}^{N-1}y_{n}^{2}$. Therefore, the sensing node adopts the energy detection method. Next, R is used to represent the judgment domain about *T* where the violation behavior exists, which is equivalent to the judgment domain about y.

However, due to various factors, there is no prior information on abnormal behavior, and the key parameters, $\sigma_{2}^{2}$, are unknown consequently. Therefore, for the detection problem of a lack of prior information, the generalized likelihood ratio test is a solution.

First of all, the maximum likelihood estimation of the unknown parameter $\sigma_{2}^{2}$ is obtained through $\sigma_{2}^{2}=\underset{\sigma_{2}^{2}}{\arg\max}p\left(y;\sigma_{2}^{2}\right)=\sum_{n=0}^{N-1}y_{n}^{2}/N$, which can be substituted into Equation (12) to obtain:

(13) $$g_{GLRT}\left(T\right)=\sum_{i\in \{0,1\}}\lambda_{i}T^{\frac{N}{2}}\exp\left(-\frac{T}{2\sigma_{i}^{2}}\right)<1,$$

where $\lambda_{i}=\rho_{i}{\left(\frac{e}{N\sigma_{i}^{2}}\right)}^{\frac{N}{2}}$, due to the presence of abnormal power, $\sigma_{2}^{2}>\sigma_{0}^{2}$. Therefore, in R, $T>N\sigma_{0}^{2}$. Hence, the judgment domain can be expressed as:

(14) $$R=\left\{T|\sum_{i\in \{0,1\}}\lambda_{i}T^{\frac{N}{2}}\exp\left(-\frac{T}{2\sigma_{i}^{2}}\right)<1,T>N\sigma_{0}^{2}\right\},$$

where $\lambda_{i}$ is determined by the false alarm constraint in the target function. As a result, the result of the judgment on the abnormal behavior can be expressed as $d=\left\{\begin{array}{cc} 1 & T\in R\\ 0 & T\notin R \end{array}\right.$, where d=1 indicates the existence of abnormal transmission power.

When $H_{i}$ is true, the test statistics obey the Chi-Square distribution: $T=\sum_{n=0}^{N-1}y_{n}^{2}∼\chi_{N}^{2}\left(\sigma_{i}^{2}\right)$. Therefore, the detection probability can be derived from the following equation:

(15) $$\begin{array}{c} \mathrm{Pr}\left(H_{2}|H_{i}\right)=\int_{y\in A}\frac{1}{{\left(2\pi \sigma_{i}^{2}\right)}^{\frac{N}{2}}}\exp\left(-\frac{\sum_{n=0}^{N-1}y_{n}^{2}}{2\sigma_{i}^{2}}\right)dy\\ =\int_{T\in R}\frac{1}{2^{\frac{N}{2}}\Gamma \left(\frac{N}{2}\right)}{\left(\frac{T}{\sigma_{i}^{2}}\right)}^{\frac{N}{2}-1}\exp\left(-\frac{T}{2\sigma_{i}^{2}}\right)dT. \end{array}$$

By introducing equation $g_{i}\left(T\right)=\lambda_{i}T^{\frac{N}{2}}\exp\left(-\frac{T}{2\sigma_{i}^{2}}\right),i\in \left(0,1\right)$, the derivative with respect to *T* can be: when $T>N\sigma_{i}^{2}$, $g{}_{i}^{\prime }\left(T\right)>0$, otherwise $g{}_{i}^{\prime }\left(T\right)\leq 0$. Therefore, there a $\eta_{2}$ that makes $g_{0}\left(\eta_{2}\right)+g_{1}\left(\eta_{2}\right)=1$, i.e., $\left\{T|T>\eta_{2}\right\}\in R$. Meanwhile, in the region $\left(N\sigma_{0}^{2},N\sigma_{1}^{2}\right)$, $g{}_{0}^{\prime }\left(T\right)>0$, $g{}_{1}^{\prime }\left(T\right)<0$. This region may partly belong to the decision domain, but it’s closely related to the SNR under normal conditions, i.e., $\frac{\sigma_{1}^{2}-\sigma_{0}^{2}}{\sigma_{\omega }^{2}}$. Specifically, this part of the decision domain can be divided into two situations:

- When the SNR is small, $R=\left[\eta_{2},\infty \right)$. At this time, the introduced function $\sum_{i\in [0,1]}g_{i}\left(T\right)=1,T>N\sigma_{0}^{2}$ only holds one solution. Therefore, $\mathrm{Pr}\left(H_{2}|H_{i}\right)=\mathrm{Pr}\left(T>\eta_{2}|H_{i}\right)=\frac{\Gamma \left(\frac{N}{2},\frac{\eta_{2}}{\sigma_{i}^{2}}\right)}{\Gamma \left(\frac{N}{2}\right)}\leq \alpha_{i}$. To satisfy two false alarm constraints, $\eta_{2}=\max\left(\Gamma^{-1}\left(\frac{N}{2},\left(\alpha \right)\Gamma \left(\frac{N}{2}\right)\right)\sigma_{0}^{2},\Gamma^{-1}\left(\frac{N}{2},\beta \Gamma \left(\frac{N}{2}\right)\right)\sigma_{0}^{2}\right)$.
- When the SNR is large, $R=\left[\eta_{0},\eta_{1}\right]\cup \left[\eta_{2},\infty \right)$, $N\sigma_{0}^{2}<\eta_{0}<\eta_{1}<N\sigma_{1}^{2}<\eta_{2}$, where $\eta_{0},\eta_{1},\eta_{2}$ are the three solutions of the introduced function $\sum_{i\in [0,1]}g_{i}\left(T\right)=1,T>N\sigma_{0}^{2}$. At this point, the two false alarm probabilities $\mathrm{Pr}(\left.H_{2}|H_{0}\right)$ and $\mathrm{Pr}\left(H_{2}|H_{1}\right)$ reach the upper limit, respectively, which satisfies $\mathrm{Pr}(\left.H_{2}|H_{0}\right)=\alpha_{0},\mathrm{Pr}\left(H_{2}|H_{1}\right)=\alpha_{1}$.

#### 3.2.2. Multi-Node Detection under Heterogeneous False-Alarm Constraint

To further improve detection performance, this subsection considers the collaborative detection of multiple nodes. Specifically, after *N* nodes make their decisions, the decision results will be reported to the fusion center, which will conduct data fusion and further give a global decision. At this time, aiming at weakening the influence of the PU-T on the sensing data, the node selection mechanism based on SINR is chosen because SINR can well reflect the influence of PU-T on the sensing data. Specifically, the SINR at the k-th sensing node can be represented as:

(16) $$SINR=\frac{p_{1}{\left(\frac{d_{1}}{d_{1,k}}\right)}^{\alpha }}{p_{0}{\left(\frac{d_{0}}{d_{0,k}}\right)}^{\alpha }+W_{k}},$$

where α is the path fading factor, $d_{1}$ is the reference distance, $p_{1}$ is the estimated power at the reference distance, $d_{1,k}$ is the distance from the secondary user to the k-th node and $W_{k}$ is the noise power at the k-th node. Therefore, the set of the sensing nodes which participate in the global decision can be obtained by:

(17) $$K=\left\{k|\frac{1}{P_{0,k}+W_{k}}{\left(\frac{d_{0}}{d_{1,k}}\right)}^{\alpha }>\chi \right\},$$

where the threshold χ is a changeable constant and the value of χ can be adjusted slightly according to the detection performance. Further, the ‘L out of K’ is adopted as a decision rule, i.e., $\sum_{k=1}^{K}d_{k}\overset{D=1}{\underset{D=0}{\frac{>}{<}}}L$, with $d_{k}\in \left\{0,1\right\}$ represents the decision result of the k-th sensing node, and L is the global decision threshold.

Consistent with single-node detection, multi-node detection adopts the similar detection criteria, namely maximizing detection probability under the constraint of global false alarm probability:

(18) $$\begin{array}{l} \max_{L}\mathrm{Pr}\left(D=H_{2}|H_{2}\right)\\ s.t.\mathrm{Pr}\left(D=H_{2}|H_{0}\right)\leq \beta_{0},\mathrm{Pr}\left(D=H_{2}|H_{1}\right)\leq \beta_{1}, \end{array}$$

where $\beta_{0}$ and $\beta_{1}$ are global false alarm probability constraints, $0<\beta_{0}\leq \alpha_{0},0<\beta_{1}\leq \alpha_{1}$. When the distance between nodes is much less than the distance between nodes and users, the detection performance of each node is close. Therefore, assuming that the performance of each node is the same, the false alarm constraint can be expressed as:

(19) $$\mathrm{Pr}\left(D=H_{2}|H_{i}\right)=\sum_{l=L}^{K}C_{K}^{l}{\left(\mathrm{Pr}\left(H_{2}|H_{i}\right)\right)}^{l}{\left(1-\mathrm{Pr}\left(H_{2}|H_{i}\right)\right)}^{K-l}\leq \beta_{i}.$$

From the above equation, when the global decision threshold increases, the two false alarm probabilities increase, and the global detection probability also increases. Therefore, to maximize the detection probability, the optimal global decision threshold should be set to the minimum value satisfying two constraints. Therefore, Equation (19) can be solved respectively, which can be further simplified as $L\geq L_{0},L\geq L_{1}$, where $L_{0}$ and $L_{1}$ is the minimum solution of the equation when i=0,1, respectively. Finally, the larger one will be chosen as the global decision threshold.

### 3.3. Detection of TPV at Multiple Power Levels

Different from the heterogeneous false alarm constraint in Equation (8) in the previous subsection, the Neyman–Pearson criterion is used in this section, that is, maximizing detection probability under the constraint of false alarm probability. Specifically, the criterion can be expressed as follows:

(20) $$\begin{array}{l} \max\mathrm{Pr}\left(O_{1}|O_{1}\right)\\ s.t.\mathrm{Pr}\left(O_{1}|O_{0}\right)\leq \alpha , \end{array}$$

where $\mathrm{Pr}\left(O_{1}|O_{0}\right)=\sum_{l=0}^{L}\mathrm{Pr}\left(O_{1}|H_{l}\right)\mathrm{Pr}\left(H_{l}|O_{0}\right)$, α represents the false-alarm constraint.

#### 3.3.1. Generalized Maximum Likelihood Detection

For the above problems, the likelihood ratio detection is derived as follows:

(21) $$\frac{p\left(y|O_{1}\right)}{p\left(y|O_{0}\right)}=\frac{\int_{P_{a}\notin \left\{P_{t}^{\left(0\right)},{P_{t}}^{{}_{\left(1\right)}},\ldots ,P_{t}^{\left(L\right)}\right\}}p\left(y|P_{a}\right)p\left(P_{a}|O_{1}\right)dP_{a}}{\sum_{l=0}^{L}p\left(y|P_{t}^{\left(l\right)}\right)\mathrm{Pr}\left(P_{t}^{\left(l\right)}|O_{0}\right)}\overset{O_{1}}{\underset{O_{0}}{\frac{>}{<}}}\lambda_{B},$$

where $p\left(P_{a}|O_{1}\right)$ is the probability density function of abnormal power, $\mathrm{Pr}\left(P_{t}^{\left(l\right)}|O_{0}\right)$ denotes the conditional distribution of normal channel state, and the value of decision threshold $\lambda_{B}$ is determined by the false alarm constraint in Equation (20). The temporary symbol $P_{r}$ is introduced to represent any transmitted power, and the conditional probability of the sample under the transmitted power is expressed as follows:

(22) $$p\left(y|P_{r}\right)=\frac{1}{{\left[2\pi \left(h_{1,k}^{2}P_{r}+\sigma_{0}^{2}\right)\right]}^{\frac{M}{2}}}\exp\left[-\frac{\sum_{m=1}^{M}y^{2}\left(m\right)}{2\left(h_{1,k}^{2}P_{r}+\sigma_{0}^{2}\right)}\right].$$

Replace $P_{r}$ with $P_{a}$ and $P_{t}^{\left(l\right)}$, and substitute them into Equation (21) respectively, the test statistics can be obtained as $T=\sum_{m=1}^{M}y^{2}\left(m\right)$. This means that the energy detector is optimal for detecting emission power anomalies when the information is fully known. However, the probability density function of abnormal power $p\left(P_{a}|O_{1}\right)$ is often unknown, so the detector mentioned above cannot be realized. Hence, a more practical detection method needs to be designed.

To solve the problem of detecting abnormal transmission power under unknown distribution, the generalized likelihood ratio (GLR) detector is derived by maximum likelihood estimation of unknown parameters in this section. Firstly, we can take the derivative of $p\left(y|P_{a}\right)$ with respect to $P_{a}$, and the maximum likelihood estimation of $P_{a}$ can be obtained:

(23) $${\hat{P}}_{a}=\arg\max_{P_{a}}p\left(y|P_{a}\right)=\frac{T/M-\sigma_{0}^{2}}{h_{1,k}^{2}}.$$

And then we can get

(24) $$\begin{array}{c} \frac{p\left(y|{\hat{P}}_{a},O_{1}\right)}{p\left(y|O_{0}\right)}=\frac{\frac{1}{{\left[2\pi \left(h_{1,k}^{2}{\hat{P}}_{a}+\sigma_{n}^{2}\right)\right]}^{\frac{M}{2}}}\exp\left[-\frac{\sum_{m=1}^{M}y^{2}\left(m\right)}{2\left(h_{1,k}^{2}{\hat{P}}_{a}+\sigma_{n}^{2}\right)}\right]}{\sum_{l=0}^{L}\frac{\mathrm{Pr}\left(P_{t}^{\left(l\right)}|O_{0}\right)}{{\left[2\pi \left(h_{1,k}^{2}P_{t}^{\left(l\right)}+\sigma_{0}^{2}\right)\right]}^{\frac{M}{2}}}\exp\left[-\frac{\sum_{m=1}^{M}y^{2}\left(m\right)}{2\left(h_{1,k}^{2}P_{t}^{\left(l\right)}+\sigma_{0}^{2}\right)}\right]}\\ =\frac{1}{\sum_{l=0}^{L}{\left[\frac{T/M}{h_{1,k}^{2}P_{t}^{\left(l\right)}+\sigma_{0}^{2}}\exp\left(1-\frac{T/M}{h_{1,k}^{2}P_{t}^{\left(l\right)}+\sigma_{0}^{2}}\right)\right]}^{\frac{M}{2}}\mathrm{Pr}\left(P_{t}^{\left(l\right)}|O_{0}\right)}. \end{array}$$

By introducing $C\left(T,P_{t}^{\left(l\right)}\right)=\frac{T/M}{h_{1,k}^{2}P_{t}^{\left(l\right)}+\sigma_{0}^{2}}$, the GLR detector can be expressed as

(25) $$\sum_{l=0}^{L}{\left\{C\left(T,P_{t}^{\left(l\right)}\right)e^{1-C\left(T,P_{t}^{\left(l\right)}\right)}\right\}}^{\frac{M}{2}}\mathrm{Pr}\left(P_{t}^{\left(l\right)}|O_{0}\right)\overset{O_{0}}{\underset{O_{1}}{\frac{>}{<}}}\lambda_{G}.$$

It should be noted that the detector can achieve the optimization objective in Equation (20) approximately optimally, however, it is difficult to carry out theoretical analysis due to the existence of the sum of several higher-order components.

#### 3.3.2. The AaN Detector

To simplify the GLR detector, a detector that is easy to analyze theoretically is designed. This subsubsection proposes a two-step detector, the AaN detector, whose core idea is to first select the most likely normal state, and then determine whether there is abnormal behavior.

Specifically, in the first step, the maximum a posterior (MAP) criterion is adopted to select the most likely one from multiple normal power levels, which can be expressed as:

(26) $$\begin{array}{c} P_{t}^{\left(l_{0}\right)}=\underset{P_{t}^{\left(l\right)}}{\arg\max}p\left(P_{t}^{\left(l\right)}|y,O_{0}\right)\\ =\underset{P_{t}^{\left(l\right)}}{\arg\max}p\left(y|P_{t}^{\left(l\right)}\right)\mathrm{Pr}\left(P_{t}^{\left(l\right)}|O_{0}\right). \end{array}$$

In the second step, the likelihood ratio between the normal state and the abnormal state is derived. The power in the abnormal state is estimated by Equation (23), and the power in the normal state is the power level selected by Equation (26). Specifically, the likelihood ratio is expressed as follows:

(27) $$\begin{array}{c} \frac{p\left(y|{\hat{P}}_{a},O_{1}\right)}{p\left(y|P_{t}^{\left(l_{0}\right)}\right)\mathrm{Pr}\left(P_{t}^{\left(l_{0}\right)}|O_{0}\right)}=\frac{p\left(y|{\hat{P}}_{a},O_{1}\right)}{\max_{l\in \{0,1,\ldots L\}}p\left(y|P_{t}^{\left(l\right)}\right)\mathrm{Pr}\left(P_{t}^{\left(l\right)}|O_{0}\right)}\\ =\frac{1}{\max_{l\in \{0,1,\ldots L\}}{\left[\frac{T/M}{h_{1,k}^{2}P_{t}^{\left(l\right)}+\sigma_{0}^{2}}e^{\left(1-\frac{T_{l}/M}{h_{1,k}^{2}R_{t}^{\left(l\right)}+\sigma_{0}^{2}}\right)}\right]}^{\frac{M}{2}}\mathrm{Pr}\left(P_{t}^{\left(l\right)}|O_{0}\right)}. \end{array}$$

We also have:

(28) $$\begin{array}{c} \max_{l\in \{0,1,\ldots L\}}{\left\{C\left(T,P_{t}^{\left(l\right)}\right)e^{1-C\left(T,P_{t}^{\left(l\right)}\right)}\right\}}^{\frac{M}{2}}\mathrm{Pr}\left(P_{t}^{\left(l\right)}|O_{0}\right)\\ =\max_{l\in \{0,1,\ldots L\}}{\left\{\begin{array}{c} C\left(T,P_{t}^{\left(l\right)}\right)e^{1-C\left(T,P_{t}^{\left(l\right)}\right)}\cdot {\left[\mathrm{Pr}\left(P_{t}^{\left(l\right)}|O_{0}\right)\right]}^{\frac{2}{M}} \end{array}\right\}}^{\frac{M}{2}}\overset{O_{0}}{\underset{O_{1}}{\frac{>}{<}}}\lambda_{m}. \end{array}$$

As $C\left(T,P_{t}^{\left(l\right)}\right)>0$, the AaN detector is finally expressed as:

(29) $$\max_{l\in \{0,1,\ldots L\}}C\left(T,P_{t}^{\left(l\right)}\right)e^{\left[1-C\left(T,P_{t}^{\left(l\right)}\right)\right]}p_{0}^{\left(l\right)}\overset{O_{0}}{\underset{O_{1}}{\frac{>}{<}}}\lambda_{m},$$

where $p_{0}^{\left(l\right)}={\left[\mathrm{Pr}\left(P_{t}^{\left(l\right)}|O_{0}\right)\right]}^{\frac{2}{M}},\;\lambda_{M}=\lambda_{m}^{\frac{2}{M}}$. In particular, in the AaN detector, this subsubsection simplifies the expression of detector through two-step decomposition, thus making detection performance analysis possible. Furthermore, based on this, the threshold can be set more reasonably.

#### 3.3.3. Threshold Setting

**Figure 3:**
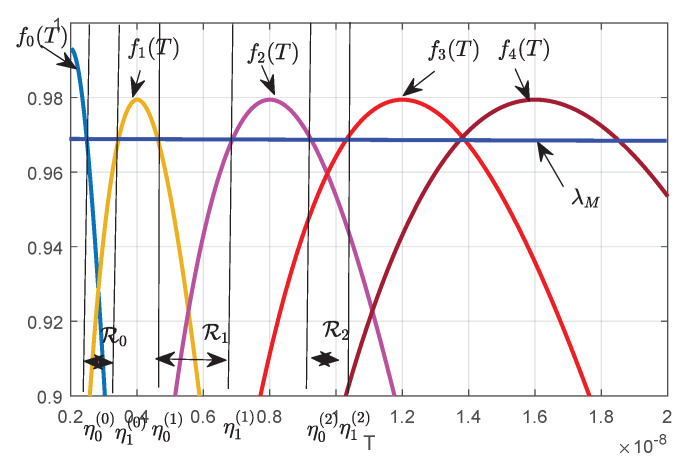
The decision field of AaN detector ($P_{a}=1\;W$), which is divided into N+1 small intervals due to the existence of multiple normal power levels.

By introducing $f_{l}\left(T\right)=C\left(T,P_{t}^{\left(l\right)}\right)\exp\left[1-C\left(T,P_{t}^{\left(l\right)}\right)\right]p_{0}^{\left(l\right)}$, the detector in Equation (29) can be re-expressed as $F\left(T\right)=\max_{l\in \{0,1,\ldots L\}}f_{l}\left(T\right)\overset{O_{0}}{\underset{O_{1}}{\frac{>}{<}}}\lambda_{M}$. Further, as shown in Figure 3, due to the existence of multiple normal power levels, the decision domain is divided into N+1 small intervals as follows:

(30) $$R=R_{0}\cup R_{1}\cup \ldots \cup R_{N},$$

where $R_{n}=\left\{T|F\left(T\right)\leq \lambda_{M},F\left(\eta_{0}^{\left(n\right)}\right)=F\left(\eta_{1}^{\left(n\right)}\right)=\lambda_{M},n=0,1,2,\ldots ,N,\eta_{0}^{\left(n\right)}\leq T\leq \eta_{1}^{\left(n\right)}\right\},\eta_{1}^{\left(n\right)}<\eta_{0}^{\left(n+1\right)}$ and $R_{i}\cup R_{j}=\emptyset ,n,i,j\in \left\{0,1,\ldots ,N\right\},i\neq j$. Further, there are three special statements about the decision domain as follows:

- $\eta_{0}^{\left(n\right)}>M\sigma_{0}^{2}$, when the terrestrial user is working, its actual transmitted power $P_{r}>0$. The variance of the observed quantity $y_{k}\left(m\right)$ must be greater than $\sigma_{0}^{2}$, so the estimated variance of the observed data should be greater than $M\sigma_{0}^{2}$;
- $\eta_{1}^{\left(N\right)}=\infty$, because when $T>M\left(h_{1,k}^{2}P_{t}^{\left(l\right)}+\sigma_{0}^{2}\right)$, $f_{l}\left(T\right)$ is monotonically decreasing. Therefore, there is $T_{u}>M\left(h_{1,k}^{2}P_{t}^{\left(l\right)}+\sigma_{0}^{2}\right)$ such that for all $T>T_{u}$, it satisfies $F\left(T\right)<\lambda_{M}$, i.e., $O_{1}$ is true.
- *N* is determined by the false alarm constraint.

As $T|P_{t}^{\left(l\right)}∼\chi_{M}^{2}\left(\sigma_{l}^{2}\right)$, where $\sigma_{l}^{2}=\sigma_{0}^{2}+h_{1,k}^{2}P_{t}^{\left(l\right)}$. Therefore, the detection probability can be obtained from the following equation:

(31) $$\begin{array}{c} \mathrm{Pr}\left(O_{1}|O_{1}\right)=\sum_{n=0}^{N}\int_{R_{n}}p\left(T|O_{1}\right)dT\\ =\sum_{n=0}^{N}\left(\frac{\Gamma \left(\frac{M}{2},\frac{\eta_{0}^{\left(n\right)}}{\sigma_{a}^{2}}\right)}{\Gamma \left(\frac{M}{2}\right)}-\frac{\Gamma \left(\frac{M}{2},\frac{\eta_{1}^{\left(n\right)}}{\sigma_{a}^{2}}\right)}{\Gamma \left(\frac{M}{2}\right)}\right), \end{array}$$

where $\sigma_{a}^{2}=\sigma_{0}^{2}+h_{1,k}^{2}P_{a}^{\left(l\right)}$. The false alarm probability can be obtained by Equation (32):

(32) $$\begin{array}{c} \mathrm{Pr}\left(O_{1}|O_{0}\right)=\sum_{n=0}^{N}\int_{R_{n}}\sum_{l=1}^{L}p\left(T|P_{t}^{\left(l\right)},O_{0}\right)\mathrm{Pr}\left(P_{t}^{\left(l\right)}|O_{0}\right)dT\\ =\sum_{n=0}^{N}\sum_{l=1}^{L}\left(\frac{\Gamma \left(\frac{M}{2},\frac{\eta_{0}^{\left(n\right)}}{\sigma_{l}^{2}}\right)}{\Gamma \left(\frac{M}{2}\right)}-\frac{\Gamma \left(\frac{M}{2},\frac{\eta_{1}^{\left(n\right)}}{\sigma_{l}^{2}}\right)}{\Gamma \left(\frac{M}{2}\right)}\right)\mathrm{Pr}\left(P_{t}^{\left(l\right)}|O_{0}\right). \end{array}$$

Next, to understand the inner mechanism of the detector more clearly and deeply, an approximate estimation of the AaN detector is made based on the following points:

- $T=M\left(h_{1,k}^{2}P_{t}^{\left(l\right)}+\sigma_{0}^{2}\right)$ is the peak point of $f_{l}\left(T\right)$, where ${f_{l}}^{\prime }\left(T\right)=0$, $f_{l}\left(T\right)=P_{0}^{\left(l\right)}$;
- When the number of samples *M* is large enough, $p_{0}^{\left(l\right)}={\left[\mathrm{Pr}\left(P_{t}^{\left(l\right)}|O_{0}\right)\right]}^{\frac{2}{M}}$ approaches 1;
- The distance between each power level is always reasonably set so that each power level can be distinguished. Finally, between the two adjacent peak points, namely, in the interval $D_{l}=\left[M\left(h_{1,k}^{2}P_{t}^{\left(l\right)}+\sigma_{n}^{2}\right),M\left(h_{1,k}^{2}P_{t}^{\left(l+1\right)}+\sigma_{n}^{2}\right)\right]$, the detector can be approximated as
  (33) $$\max_{k\in \{0,1,\ldots L\}}C\left(T,P_{t}^{\left(k\right)}\right)\exp\left[1-C\left(T,P_{t}^{\left(k\right)}\right)\right]p_{0}^{\left(k\right)}\overset{O_{0}}{\underset{O_{1}}{\frac{>}{<}}}\lambda_{m}.$$

The decision field is given by Theorem 1:

**Theorem 1.** The intersection of decision field and interval $D_{l}$ cannot be an empty set, which is expressed as: $R^{*}=\left[\eta_{0}^{*},\eta_{1}^{*}\right]$, if and only if

(34) $$F_{0}^{\left(l\right)}=C\left(T_{0}^{\left(l\right)},P_{t}^{\left(l\right)}\right)\exp\left[1-C\left(T_{0}^{\left(l\right)},P_{t}^{\left(l\right)}\right)\right]p_{0}^{\left(l\right)}<\lambda_{M},$$

*where $T_{0}^{\left(l\right)}=M\sigma_{l}^{2}\sigma_{l+1}^{2}\frac{\ln\sigma_{l+1}^{2}-\ln\sigma_{l}^{2}}{\sigma_{l+1}^{2}-\sigma_{l}^{2}}\ln\frac{p_{0}^{\left(l\right)}}{p_{0}^{\left(l+1\right)}}$, $\sigma_{l}^{2}=h_{1,k}^{2}P_{t}^{\left(l\right)}+\sigma_{n}^{2}$, hence, the two thresholds can be expressed as:*

(35) $$\left\{\begin{array}{c} \eta_{0}^{*}=\left(h_{1,k}^{2}P_{t}^{\left(l\right)}+\sigma_{0}^{2}\right)M\cdot x_{1},\\ \eta_{1}^{*}=\left(h_{1,k}^{2}P_{t}^{\left(l+1\right)}+\sigma_{0}^{2}\right)M\cdot x_{0}, \end{array}\right.$$

*where, $x_{0}$ is the smaller solution to $xe^{1-x}p_{0}^{\left(l+1\right)}=\lambda_{M}$, and $x_{1}$ is the larger solution to $xe^{1-x}p_{0}^{\left(l\right)}=\lambda_{M}$.*

## 4. Simulation Results

As for the simulations, the model of the GEO satellite refers to refer to Astra system and O3b (Other 3 billion) system. Specifically, the Equivalent isotropically radiated power (EIRP) of the satellite is set to be 62.7 dBW. The transmitted power of the satellite is set to be 37.15 W. The center frequency is 18.48 GHz. The path fading factor α=3 in this subsection, which is usually between 2 and 4. The received noise power spectral density is –174 dBm/Hz, as shown in Table 1.

### 4.1. Performance of TPV Detection with Power Level L = 1

**Figure 4:**
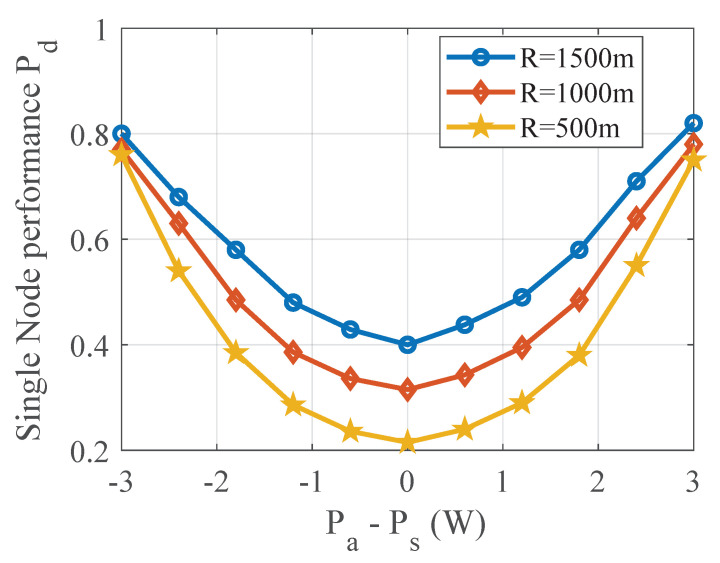
Performance of TPV detection with a single node, which demonstrates that power deviations is the greatest factor.

In this subsection, the detection probabilities of secondary users at different distances and power deviations are given. As shown in Figure 4, the detection performance is gradually improved as the distance between the SU and the PU-R becomes larger and larger. This is because of the greater the distance, the less the sensing data affected by the PU-T. At the same time, the detection performance is improved when the power deviation is gradually larger. This is because the larger the deviation, the larger the difference between the observed variance in the normal state of the model and the observed variance in the abnormal state, therefore, the detection performance will be improved. Noted that when the power deviation is large or small enough, the detection performance of different distances is very similar, and the fundamental reason is the variance difference between the observed data.

**Figure 5:**
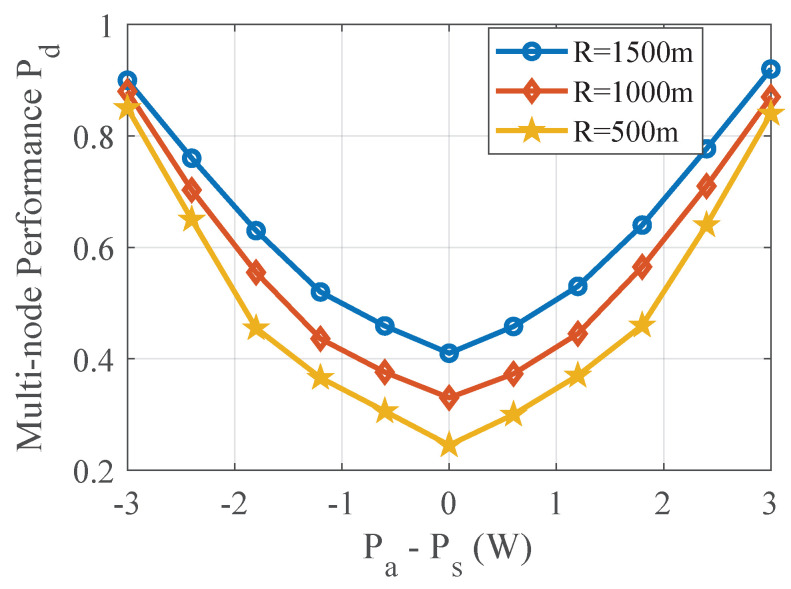
Performance of TPV detection with multiple nodes, which demonstrates that the “L out of K" can improve the performance by 5.6%.

To reduce the influence of the primary user on the sensing data, the node selection scheme based on the selection mechanism of the SINR is proposed. As shown in Figure 5, the global detection performance is improved compared with Figure 4, indicating the effectiveness of the proposed scheme. What’s more, it also shows that the power deviation, namely the variance difference, is the most important factor which affects the detection performance. However, according to the simulation results, we find that the probability increases by 4.5% when R = 1500 m, 4.8% when R = 1000 m and 7.4% when R = 500 m, comparing to the Figure 4. Noted that the increased detection probability decreases when the distance between PU-T and SU-T increases, this is because the smaller the distance, the greater the impact on the sensing data, which also indicates the superiority of the node selection scheme.

**Figure 6:**
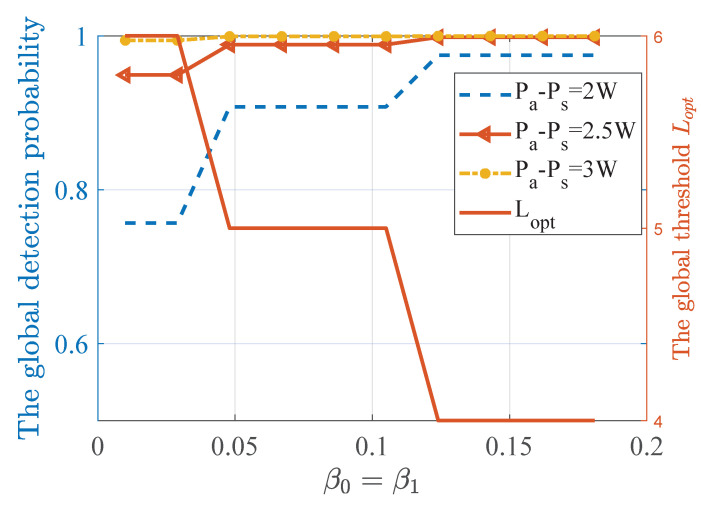
Global detection performance under different false-alarm constraints, which can reflect the compromise between false alarm probability and detection probability.

Figure 6 depicts the changing relationship between global detection probability and the global decision threshold under different false alarm constraints. Similar to the conclusion obtained by Figure 4 and Figure 5, the detection probability is positively correlated with the power difference. With the weakening of false alarm constraint, the global decision threshold increases as well as the global detection probability, which well reflects the compromise between false alarm probability and detection probability. However, due to the limited number of sensing nodes, the discrete value of the global detection threshold has no obvious change in some intervals.

### 4.2. Performance of TPV Detection with Multiple Power Levels.

**Figure 7:**
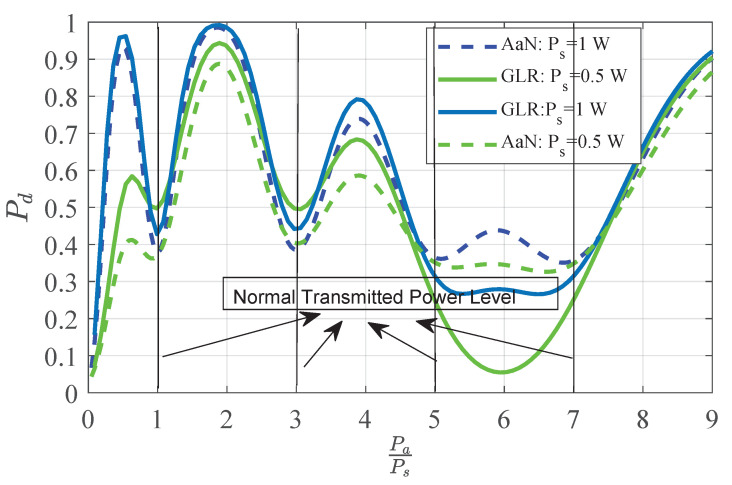
Detection probability under multiple power levels. The AaN detector can well replace the GLR detector in most intervals at the expense of less than 10% detection probability.

Figure 7 shows the detection probability under different abnormal power levels $P_{a}$, in which, the level of transmitting power level is 4, and the false alarm constraint is 0.2. As shown in Figure 7, when the benchmark transmitting power increases, the performance of the detector is improved because it is easy to distinguish the normal power under different states. At the same time, it can be noted that when $P_{a}$ falls between two adjacent normal power levels, the greater the deviation of $P_{a}$ from the nearest normal power level, the more likely it is to be distinguished from the normal state. Therefore, the detection performance is improved. However, the exception occurs when $\frac{P_{a}}{P_{s}}\in \left[5,7\right]$, the GLR detector shows an opposite trend. This is because according to theorem 1, the intersection of decision domain and interval $D_{l}$ is empty or very small. In addition, when the deviation of abnormal transmission power is certain, the detection performance deteriorates as the normal power level *L* increases from 1 to 3. When the transmitting power increases, the variance of the sensing data increases, making the minimum value $F_{0}^{\left(l\right)}$ in Equation (34) increase. Therefore, as shown in Theorem 1, the intersection between this region and the decision domain shrinks and the performance deteriorates. Moreover, by comparing the performance of the two detectors at different emission power levels, it can be found that the GLR detector shows a better performance than the AaN detector in most cases except for $\frac{P_{a}}{P_{s}}\in \left[5,7\right]$. This is because the AaN detector only considers the most likely probability level to do the likelihood ratio, which makes the test function F(T) sharper and further makes the overall performance worse and improves on some intervals. So we can come to the conclusion that the GLR detector can be replaced to some extent by the proposed detector, which can not only achieve better detection performance but also save operation cost.

**Figure 8:**
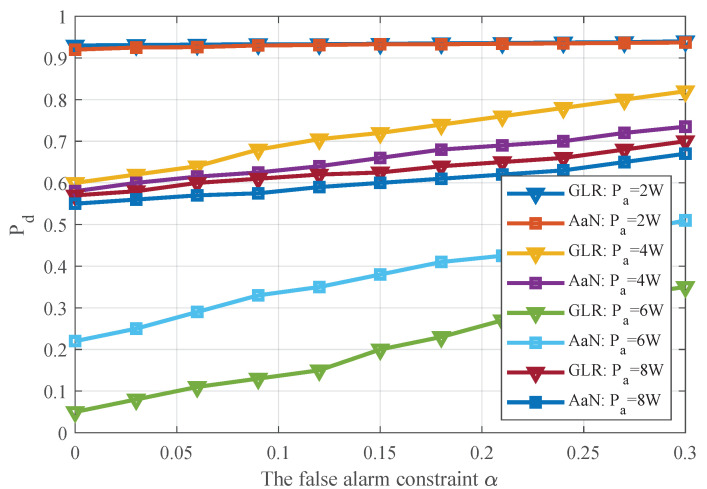
Detection performance under different false-alarm constraints, which can reflect the compromise between false alarm probability and detection probability.

Figure 8 shows the receiver operating characteristic (ROC) curve of the two detectors. It can be noted that with the enhancement of false alarm constraint, the detection probability also increases. Moreover, in most cases, the GLR detector shows a better detection performance than the AaN detector. However, as mentioned in Figure 6, when $\frac{P_{a}}{P_{s}}\in \left[5,7\right]$, the two detectors perform in the opposite way. This is also because the intersection of the decision field and interval $D_{l}$ is small or even empty.

## 5. Conclusions

In this paper, we investigate the detection problem of TPV in satellite-terrestrial integrated networks via the geolocation spectrum database based detection framework, in which spectrum usage application, allocation information, spectrum monitoring requirements, and monitoring results are exchanged. Considering the uncertainty of the normal working state, the binary hybrid hypothesis testing model is established to identify abnormal transmitting power behavior. Based on this model, the single-node detection and multi-node cooperative detection methods under heterogeneous false alarm constraints are firstly derived. Then, the detection problem of TPV under multi-power levels is considered. To simplify the theoretical analysis in the scenario of TPV under multi-power levels, the AaN detector was developed. Finally, the simulation results demonstrate that the proposed detection method can not only improve the performance by nearly 5.6% in the scenario of single transmitted power level, but when it comes to multiple ones, it can approximately realize the detection of TPV in most cases at the expense of less than 10% detection probability. 

## Figures and Tables

**Table 1:** Simulation Parameters.

The Notation	Physical Meaning	Value
$f_{c}$	Frequency band	18.48GHz
*h*	Satellite height	35,786 Km
EIRP	Satellite EIRP	62.7dBW
$L_{n}$	Desired side-lobe level relative to peak gain	−20dB
$h_{s,\max}$	Max satellite antenna gain	47dBi
α	The path fading factor	3

## Abbreviations

The following abbreviations are used in this manuscript:

TPV	transmitted power violation
GMNP	generalized multi-hypothesis Neyman–Pearson
PUEA	primary user emulation attack
PU-T	primary user’s transmitter
SU-T	secondary user’s transmitter
GLR	generalized likelihood ratio
AaN	Abnormal after Normal
